# Cost-effectiveness analysis of vitamin A supplementation delivery modalities in the DRC, Togo, and Niger: informing sustainable program design

**DOI:** 10.1093/heapol/czag032

**Published:** 2026-03-11

**Authors:** Lucia Corball, Kenya Chappel, Hannah Rowett, Arnaud Laillou, Andreas Hasman, Natsuki Kawai

**Affiliations:** Health Economics, Reforma Global, Appeldoorn, Gelderland, 7314HW, Netherlands; Human Development, Genesis Analytics (Pty) Ltd, 50 Sixth Road, Hyde Park, Johannesburg 2196, South Africa; Human Development, Genesis Analytics (Pty) Ltd, 50 Sixth Road, Hyde Park, Johannesburg 2196, South Africa; Nutrition, UNICEF West and Central Africa Regional Office, Route des Almadies, Immeuble Madjiguène, BP 29720, Dakar, Sénégal; Nutrition, UNICEF Headquarters,3 United Nations Plaza, New York, NY 10017, United States; Nutrition, UNICEF West and Central Africa Regional Office, Route des Almadies, Immeuble Madjiguène, BP 29720, Dakar, Sénégal

**Keywords:** micronutrient, economic evaluation, vitamin A supplementation, child nutrition, cost-effectiveness

## Abstract

This study examines how the costs, health impacts, and efficiency of two-dose vitamin A supplementation (VAS) vary across delivery platforms, population subgroups, and delivery contexts in Togo, Niger, and the Democratic Republic of the Congo (DRC). Using a scenario-based model, it compares provider costs and disability-adjusted life years (DALYs) averted and identifies reallocations that maximize health gains under fixed budget constraints. Costs were estimated from the health provider perspective, and health outcomes were measured in DALYs averted. The results showed that cost-effective scenarios varied significantly across countries; in the DRC and Niger, campaigns delivered high coverage but at a substantially higher cost than routine delivery, whereas in Togo, campaigns were both low-cost and high-coverage. In all countries, the most cost-effective scenarios prioritized children aged 6–23 months. More than two-thirds of the cost-effective scenarios achieved better outcomes at lower cost than countries’ current delivery strategies, highlighting significant potential for efficiency gains. A positive, nonlinear relationship between incremental costs and DALYs averted was observed: greater investment generally led to larger health gains but returns diminished as costs increased. A sensitivity analysis showed that facility-based supply shortages negatively affected effectiveness, whereas strengthened routine delivery improved effectiveness. Optimal VAS strategies must be context-specific, balancing the reach of campaigns with the sustainability of routine services. The decision tool provides a practical mechanism for identifying cost-effective delivery strategies tailored to national capacities and constraints.

Key messages

- In response to funding constraints, low- and middle-income countries are transitioning from resource-intensive campaigns to routine delivery of VAS. This study seeks to support this decision-making in Togo, Niger, and the DRC by creating a tool that suggests the most cost-effective mix of service-delivery modalities for VAS within their particular context.
- The results show that the cost-effectiveness of VAS delivery is highly context-dependent. For the DRC and Niger, where campaigns cost over twice as much as routine delivery, the most cost-effective strategies use a mix of campaigns and routine delivery tailored to urban or rural location and age group. In Togo, however, campaigns are lower cost and high coverage, making them the predominant cost-effective option.
- Targeting younger children and strengthening routine delivery systems enhances effectiveness. The most cost-effective scenarios prioritized children aged 6–23 months, who yielded the greatest health gains.
- Reliable supply chains are critical to stable delivery. A sensitivity analysis showed that supply shortages significantly reduced the effectiveness of VAS delivery by displacing cost-effective scenarios.

## Introduction

Vitamin A deficiency is a major contributor to child mortality in low- and middle-income countries, increasing vulnerability to infections such as measles and diarrhea (Imdad et al. 2022). High-dose vitamin A supplementation (VAS) for children aged 6–59 months is a long-standing, evidence-based intervention recommended by the World Health Organization (WHO). Systematic reviews show that delivering two doses annually reduces all-cause mortality by approximately 12% (Imdad et al. 2022). As a result, VAS is a core child survival. Despite its proven effectiveness and relatively low unit cost, sustaining high and equitable VAS coverage remains a persistent challenge, particularly in resource-constrained settings (Stevens et al. 2015).

Many countries, including Niger and the Democratic Republic of the Congo (DRC), remain below the WHO’s 80% two-dose VAS coverage (UNICEF 2018). In contrast, Togo has maintained comparatively high coverage levels (UNICEF 2023). These differences highlight the importance of delivery systems in shaping population-level impact.

VAS is delivered through multiple service delivery modalities, including routine health services at health facilities, community-based delivery by community health workers (CHWs), as well as campaign-based approaches. Campaigns often achieve higher coverage, particularly in hard-to-reach populations, but they are typically more resource-intensive (Gatobu et al. 2017, Horton et al. 2018). Routine delivery offers greater sustainability but faces challenges in reaching children in remote areas and those older than 24 months, who have often completed their routine immunization schedules (Janmohamed et al. 2024).

Campaigns are costly and frequently dependent on external funding. Declines in funding for polio campaigns, which have historically provided an important delivery platform for VAS, and limited integration of VAS campaigns into routine health systems pose growing risks to program continuity (Doherty et al. 2010, UNICEF 2018). Recent evidence suggests that prioritizing children aged 6–23 months may generate greater health impact and better value for money (Karlsson et al. 2022, Laillou et al. 2024). Recent optimization studies emphasize that conclusions about the relative efficiency of vitamin A strategies are highly sensitive to delivery performance and financing constraints, reinforcing the need for comparative analyses that reflect real-world conditions (Colina et al. 2025).

Against this backdrop, policy discussions increasingly focus on whether, when, and how countries can transition away from resource-intensive campaigns toward greater reliance on routine delivery, without compromising coverage or health impact. There is limited empirical evidence on how different delivery modalities perform in practice, particularly when accounting for real-world constraints. Existing modeling tools, including the Lives Saved Tool and Optima Nutrition, estimate health impacts under assumed coverage targets but do not assess the relative efficiency of alternative delivery platforms or account for operational frictions affecting realized coverage. Although Optima Nutrition supports detailed optimization analyses, it treats coverage as freely adjustable through investment rather than as constrained by modality-specific delivery performance, limiting its suitability for analyzing short-term reallocations under fixed system capacity.

To address this gap, we develop a decision-support tool to assess the cost-efficiency and cost-effectiveness of alternative VAS delivery strategies under empirically observed delivery constraints. Rather than treating coverage as a direct decision variable, the tool assigns children to delivery modalities by age group and geographic setting, and estimates realized coverage using observed modality-specific uptake rates. Costs are incurred only for children successfully reached, explicitly capturing delivery failures. The tool evaluates a wide range of feasible delivery scenarios and identifies efficient strategies using an efficiency frontier relating costs to disability-adjusted life years (DALYs).

The objective of this analysis is not to identify a single universally optimal delivery modality or to model the long-term investments required to expand system capacity or stimulate demand. Instead, the focus is on identifying efficient reallocations of the existing delivery modalities that can improve health outcomes within current system constraints and financing envelopes.

This study applies this approach to three countries with contrasting delivery contexts—Togo, Niger, and the DRC—to address four research questions: (i) How do the costs of two-dose VAS differ across delivery platforms? (ii) How do these differences translate into health outcomes measured in DALYs averted relative to current service delivery strategies? (iii) Which delivery scenarios represent the most efficient use of resources? (iv) How can delivery modalities be reallocated across population subgroups to maximize health gains for a given financing envelope? By explicitly incorporating delivery frictions, supply limitations, and differential access across populations, the analysis aims to inform more realistic and context-sensitive VAS policy decisions in an era of constrained resources and increasing emphasis on sustainability.

## Methods

**Figure 1:**
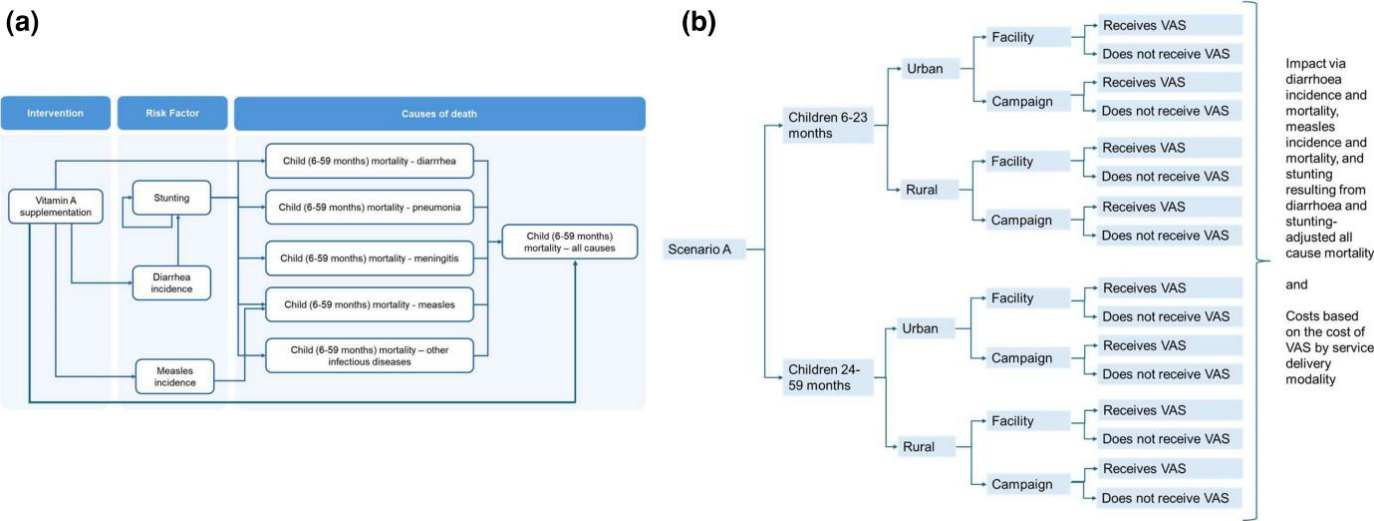
Overview of the decision-analytic model used to assess the cost-effectiveness of vitamin A supplementation delivery modalities. Panel (a) illustrates the impact pathways linking vitamin A supplementation to reductions in diarrhea incidence, measles incidence, stunting, and cause-specific mortality, which are aggregated to all-cause mortality among children aged 6–59 months. Adapted from the Lives Saved Tool (LiST) Visualizer developed by the Institute for International Programs at Johns Hopkins Bloomberg School of Public Health and maintained by Avenir Health. Panel (b) presents the decision tree structure used to model service delivery scenarios, in which children are stratified by age group (6–23 months and 24–59 months), place of residence (urban and rural), and delivery modality (facility-based or campaign-based). For each branch, children may receive or not receive vitamin A supplementation, with associated probabilities, health outcomes, and costs determined by the delivery modality.

This study applies a decision-analytic modeling approach tool to evaluate the cost-effectiveness of alternative scenarios for delivering annual, age-appropriate VAS in Togo, the DRC, and Niger. The analysis compares multiple delivery scenarios against a country-specific baseline reflecting the current VAS delivery strategies. Health outcomes are measured in DALYs averted, and program costs are estimated for each scenario. An efficiency frontier is constructed to examine the relationship between total costs and disease burden (total DALYs) across scenarios. Incremental cost-effectiveness ratios (ICERs), expressed as the cost per DALY averted, are subsequently calculated for relevant comparisons with a baseline strategy. A decision tree model (Fig. 1) is used to simulate the progression of children through alternative delivery pathways based on assigned service delivery modalities and their associated probabilities. This study is reported in accordance with the Consolidated Health Economic Evaluation Reporting Standards (CHEERS) 2022 checklist (Supplementary Material S1).

The use of artificial intelligence tools was limited to supporting the literature review (Gemini Deep Research) and assisting with language refinement (ChatGPT). All analytical work and manuscript content were reviewed, verified, and edited by the authors.

### Model overview and analytical approach

The analytical approach differs from standard cost-effectiveness models in two important ways. First, delivery modalities are treated as assignment mechanisms, not deterministic guarantees of coverage. Children assigned to a delivery platform (routine facility-based services or campaign-based delivery) are assumed to receive the annual recommended vitamin A dose (100 000 IU for those aged 6–11 months and 200 000 IU for those aged 12–59 months, delivered biannually) only with a modality-, age-, and geography-specific probability derived from empirical data. Second, costs are incurred only for children who are successfully reached, explicitly capturing losses from demand-side barriers and supply-side constraints. The model evaluates a large set of feasible delivery scenarios that reallocate service delivery modalities across age groups (6–23 months and 24–59 months) and geographic settings (urban and rural). For each scenario, the model estimates realized VAS coverage, total delivery costs, and disease burden measured in DALYs. Cost-efficiency is examined by relating total costs to total DALYs across scenarios using an efficiency frontier, whereas cost-effectiveness is assessed through ICERs, expressed as cost per DALY averted relative to the country-specific baseline delivery strategy. The baseline represents the current VAS delivery profile in each country, as observed in recent program data. Importantly, the model does not simulate the costs or effects of expanding delivery platforms beyond observed performance, such as capital investments, workforce expansion, or large-scale demand generation.

### Population, inclusion criteria, and time horizon

The target population includes children aged 6–59 months residing in the three study countries. The population is stratified by age group and geographic setting, reflecting differences in VAS delivery pathways, coverage patterns, and health impact across subgroups.

The analysis adopts a 1-year time horizon. DALYs were estimated over the lifetime of the child (Edejer and World Health Organization 2003). Future health outcomes and costs are discounted at an annual rate of 3%, in line with international guidelines for economic evaluation (Bill and Melinda Gates Foundation 2014).

### Model structure and coverage estimation

The model evaluates a set of feasible VAS delivery scenarios defined by the allocation of service delivery modalities across population subgroups. A total of 650 scenarios were generated for each country by varying the share of children assigned to facility-based and campaign-based delivery across population subgroups. Scenario generation used quartile increments (0.25) for the allocation of delivery modalities within each subgroup, with the constraint that the assigned shares summed to 100% for each population segment.

For each scenario, children were routed through a decision tree model (Fig. 1), which links modality assignment to realized coverage through empirically observed probabilities of uptake. The probability that a child receives VAS depends on the following three factors: (i) the assigned delivery modality, (ii) age group, and (iii) place of residence (urban or rural). Coverage probabilities were treated as fixed inputs and were not estimated probabilistically within the model; accordingly, the analysis does not generate sampling uncertainty around coverage estimates.

Modality-specific coverage probabilities were derived from administrative data collected from country teams at the Ministry of Health and/or international organizations, triangulated with Demographic and Health Surveys (DHS), and supplemented by expert consultation. Campaign-based delivery was assumed to reach 95% coverage of the assigned target group, whereas facility-based delivery achieved lower coverage, ranging from 18% to 25% among children aged 24–59 months and 55% to 67% for those aged 6–23 months, based on DHS data and expert interviews. These were adjusted for urban and rural variation, derived also from DHS and country-specific estimates.

The decision tree further incorporates variation in the effectiveness of VAS by age group. Importantly, scenarios vary the assignment of children to delivery platforms; realized coverage remains bounded by empirically observed uptake probabilities and does not imply that either platform can achieve 100% coverage.

DALYs were estimated using impact pathways consistent with the Lives Saved Tool (LiST). Changes in VAS coverage were translated into reductions in diarrhea incidence (15%), diarrhea mortality (12%), and measles incidence (50%) based on a study by Imdad et al. (2022). Baseline disease incidence and mortality rates were sourced from the Global Burden of Disease (GBD) Study, with population estimates from the United Nations World Population Prospects.

DALYs were calculated as the sum of years of life lost (YLL) and years lived with disability (YLD) (Rushby 2001). YLLs were estimated through a conversion of the mortality owing to each specific VAS scenario. YLLs were calculated by multiplying the number of child deaths by the life expectancy at death. Life expectancy at death was approximated as the difference between the country’s life expectancy at birth and the average age of a child’s death. The average age of death was assumed to be 1.2 years for children aged 6–23 months, and 3.5 years for children aged 25–59 months in the absence of specific data on age of death. Estimating YLD involved multiplying YLLs by the country- and age-group-specific YLD-to-YLL ratios reported in the GBD database (Claxton et al. 2015). These ratios reflect morbidity associated with downstream conditions affected by VAS, primarily diarrheal disease and measles, rather than disability attributed directly to VAD as a standalone health state, and incorporate severity distributions, duration assumptions, and comorbidity adjustments within the GBD modeling framework.

A summary of the key parameters used in the model is presented in the Supplementary Material.

### Costing approach

The costs were estimated from the health service provider perspective and reflect program-level delivery costs. The following cost components were included: (i) intervention costs, covering labor, vitamin A capsules, and other recurrent inputs directly required for VAS delivery; (ii) program costs, including administration, supervision, monitoring, training, communication, and management activities that support delivery; (iii) supply chain costs, encompassing transport, distribution, storage, stock management, and wastage; and (iv) investment costs, covering equipment and vehicles required for campaign-based or outreach delivery, which were excluded for facility-based delivery due to limited comparability and data availability.

Costs were estimated separately for facility-based and campaign-based delivery using a mixed approach, combining micro-costing (bottom-up) for specific inputs with top-down expenditure data where appropriate. Bottom-up (micro-costing) was the dominant method used for the costing of facility-based delivery, following the methodology and assumptions for the costing of VAS in the LiST Costing Tool. A mix of bottom-up and top-down methods was applied to campaign costing using reports of input quantities, aggregated expenditure data from ministries of health, UNICEF, and implementing partners such as Helen Keller International, unit costs, and population reached in specific mass events.

Detailed unit cost assumptions and results are provided in Supplementary Material.

Costs were applied proportionally to the number of children successfully reached in each scenario. Capital investments and broader health system strengthening costs required to expand facility capacity or stimulate demand beyond observed levels were intentionally excluded, as the analysis focuses on reallocating existing delivery modalities within current system constraints.

### Cost-efficiency and cost-effectiveness analysis

An efficiency frontier was constructed using total costs and total DALYs to identify strategies that minimize the number of DALYs for a given cost level. Scenarios were considered cost-efficient if no other scenario resulted in lower DALYs at the same cost. An incremental analysis was performed to compare the scenarios on the frontier to the baseline. Incremental costs refer to the additional financial resources needed to implement the frontier scenario compared to the baseline. Incremental outcomes are measured by DALYs averted, representing the reduction in disease burden achieved through the frontier scenario, compared with the baseline. The ICER was calculated by dividing the difference in costs by the difference in DALYs between the frontier scenario and the baseline. It is interpreted as USD per DALY averted. Following the recommended thresholds from the WHO, frontier scenarios with an ICER of less than three times the Gross Domestic Product (GDP) per capita per DALY averted were considered cost-effective relative to baseline (World Health Organization 2002). Scenarios with ICERs below three times GDP per capita per DALY averted were considered cost-effective (Robinson et al. 2017). Scenarios resulting in cost-savings and DALYs averted relative to baseline were considered dominant. The results of the incremental analysis were used to categorize scenarios as “More Effective but More Costly,” “More Effective and Less Costly,” or “Less Effective but Less Costly” for the ease of interpretation for a general audience.

### Scenario and sensitivity analyses

Sensitivity analyses examined how changes in key assumptions affected the composition and stability of the base-case efficiency frontier. Three sensitivity analyses were conducted: (i) supply constraints affecting facility-based delivery, (ii) integration of VAS into early childhood education platforms, and (iii) strengthening of facility-based delivery capacity. These inputs were drawn from key informant interviews and country engagement activities conducted as part of parallel analytical work on VAS delivery in the same countries. Across countries, financing constraints and the reliability of vitamin A capsule supply emerged consistently as the most critical factors influencing VAS delivery performance, particularly for facility-based services. In addition, UNICEF country teams identified opportunities for innovation through the integration of VAS into non–health facilities, such as Early Childhood Education (ECE) settings, and the potential gains from strengthening facility-based delivery capacity.

Sensitivity analyses were conducted using a scenario-based, one-way structural approach. Each key system constraint or improvement was varied independently relative to the base case while holding all other parameters constant. First, realized coverage under facility-based delivery was constrained by vitamin A capsule availability, such that expected coverage probabilities were adjusted downward when supply was insufficient. Second, an alternative delivery channel through ECE platforms was introduced, increasing the probability of receiving VAS for children aged approximately 36–59 months who were enrolled in ECE programs; the magnitude of this effect was determined by country-specific ECE coverage levels. It was assumed that at least 50% of children aged 36–59 months enrolled in ECE could be reached with VAS in the first year. This assumption was informed by evidence from early integration of health interventions into education platforms (Bundy et al. 2018). The same unit cost as for facility-based delivery was assumed for integrated VAS delivery in ECE. Third, a facility-strengthening scenario was examined in which the probability of receiving VAS conditional on assignment to facility-based delivery was increased across age groups, reflecting improvements in service readiness and performance. This analysis increased the assumed probability of reaching children aged 6–23 months to 80%, and those aged 24–59 months to 50%. For each sensitivity scenario, all delivery configurations were re-evaluated, and realized coverage, costs, DALYs, and efficiency frontiers were re-estimated. Changes in results were assessed by comparing shifts in the composition of the efficiency frontier relative to the base case.

### Data sources, parameter uncertainty, and validation

Model parameters were sourced from DHS surveys, the Global Burden of Disease study, United Nations population and life expectancy estimates, published meta-analyses, and program costing data from governments and development partners. Discrepancies across data sources, particularly between survey-based coverage estimates and administrative data, were documented and addressed through triangulation and expert validation.

## Results

This section presents the results of the cost-efficiency and cost-effectiveness analysis of VAS delivery strategies in the DRC, Niger, and Togo. The results are presented in four steps. First, we describe the base-case efficiency frontier constructed from all delivery scenarios evaluated under current delivery conditions. Second, we characterize the delivery strategies that appear on the efficiency frontier. Third, we present incremental cost-effectiveness results comparing frontier strategies to current national delivery approaches. Finally, we assess the robustness of base-case findings through scenario-based sensitivity analyses.

### Base-case analysis: efficiency frontier under current delivery conditions

**Figure 2:**
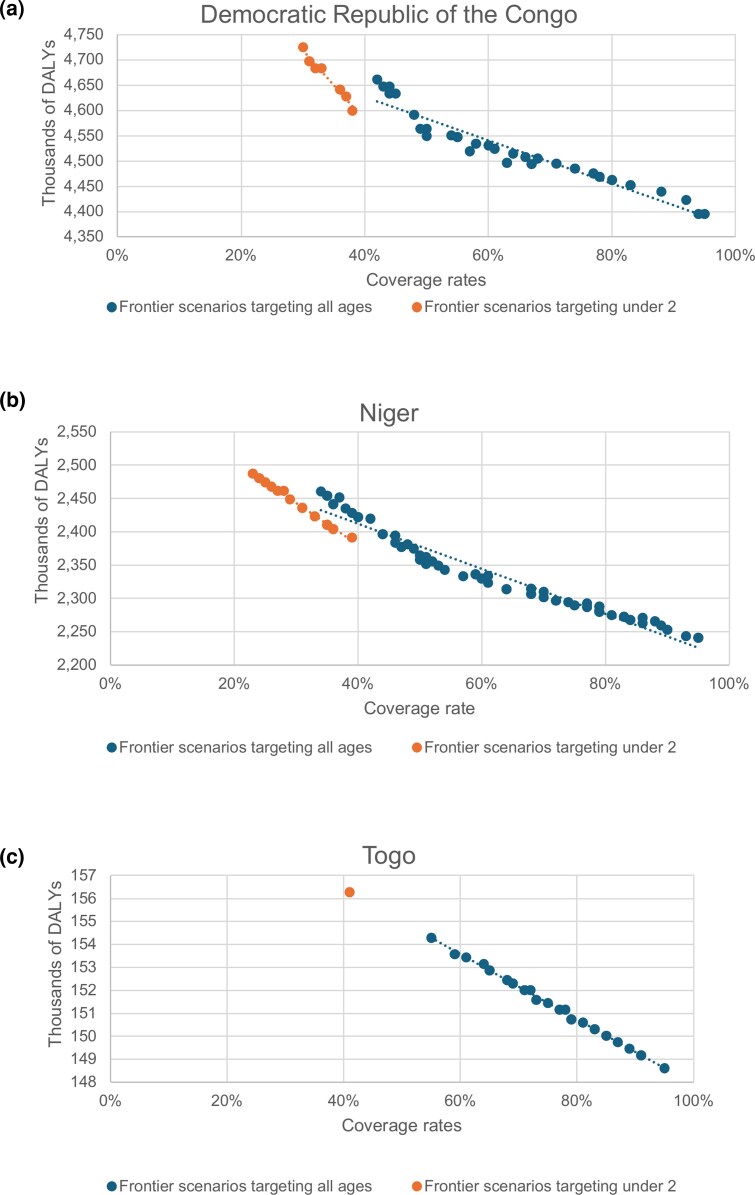
Relationship between vitamin A supplementation coverage and disability-adjusted life years (DALYs) by age-targeting strategy. The figure shows the association between vitamin A supplementation coverage rates and total DALYs among children aged 6–59 months across simulated scenarios. The results are shown separately for scenarios targeting all children aged 6–59 months and scenarios prioritizing children aged 6–23 months. Each point represents a simulated scenario, with DALYs expressed in thousands.

Examination of the efficiency frontier reveals clear patterns in the allocation of delivery modalities across population subgroups. Across countries, frontier scenarios consistently prioritized children aged 6–23 months, reflecting the higher marginal health benefits associated with VAS in this age group (Fig. 2).

Countries demonstrate varying capacities to achieve high VAS coverage under frontier scenarios (Table 1). Togo achieves the highest and most consistent coverage, with an average total coverage of 74%, including 95% among children aged 6–23 months and relatively balanced urban (76%) and rural (72%) rates. In contrast, the DRC and Niger show more variable coverage with averages of 64% and 58%, respectively.

**Table 1:** Coverage rates and costs (USD) among frontier scenarios.

	DRC			Niger			Togo		
	Average	Min	Max	Average	Min	Max	Average	Min	Max
***Coverage***									
Total	64%	30%	95%	58%	23%	95%	74%	41%	95%
6–23 months	90%	69%	95%	84%	53%	95%	95%	95%	95%
24–59 months	45%	0%	95%	38%	0%	95%	58%	0%	95%
Urban	63%	36%	94%	58%	24%	97%	76%	41%	94%
Rural	65%	27%	95%	58%	23%	95%	72%	41%	94%
***Costs (USD)***									
Baseline costs	6 108 000			3 969 000			212 000		
Costs of scenarios on the frontier	5 476 574	1 157 204	9 739 306	1 660 311	329 526	3 058 366	116 241	54 924	125 393

Table summarising vitamin A supplementation coverage rates and programme costs across cost-efficient (frontier) scenarios for the Democratic Republic of Congo (DRC), Niger, and Togo. Coverage is reported as average, minimum, and maximum values overall and by subgroup (children aged 6–23 months, 24–59 months, urban, and rural). Average total coverage ranges from 58% in Niger to 74% in Togo. Coverage among children aged 6–23 months is higher (84–95%) than among those aged 24–59 months (38–58%). Baseline programme costs are USD 6.1 million in DRC, USD 4.0 million in Niger, and USD 212,000 in Togo. Costs for frontier scenarios range from approximately USD 1.16–9.74 million in DRC, USD 0.33–3.06 million in Niger, and USD 54,924–125,393 in Togo.

The composition of delivery modalities among frontier strategies varied markedly by country. In the DRC and Niger, a substantial share of frontier scenarios employed a mixed approach, combining campaign-based delivery in rural areas with greater reliance on facility-based delivery in urban settings. In contrast, frontier strategies in Togo were predominantly campaign-based, reflecting the relatively low unit cost and high coverage achieved through campaigns in that context.

**Figure 3:**
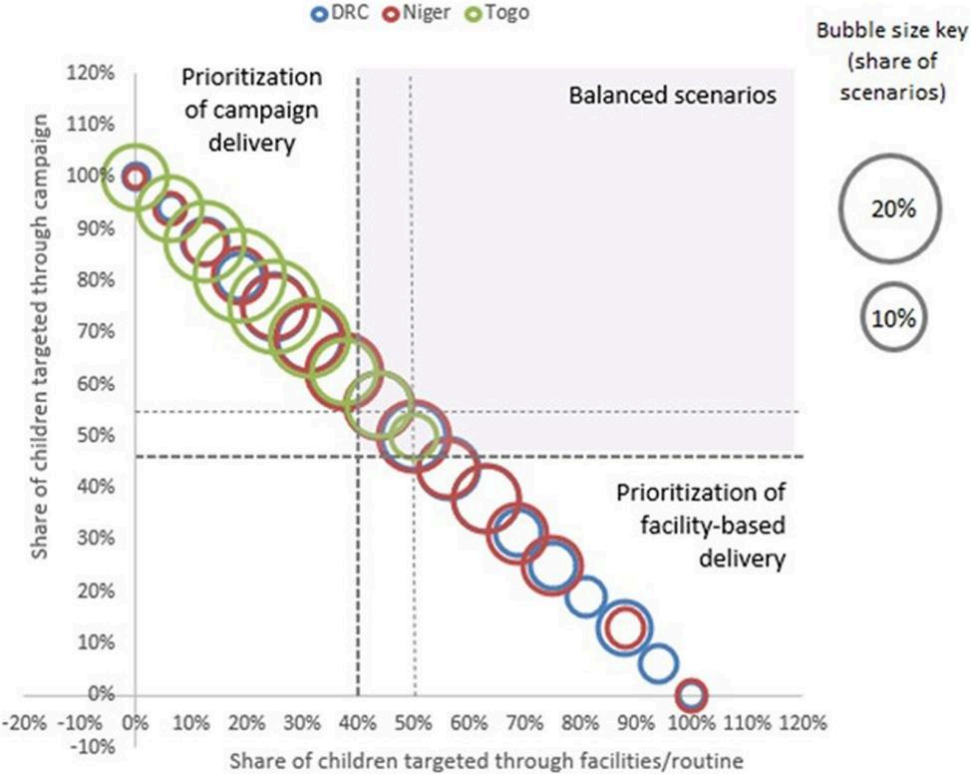
Coverage-disability-adjusted life years (DALYs) trade-offs across frontier scenarios by delivery strategy. The figure illustrates the relationship between vitamin A supplementation coverage rates and total disability-adjusted life years (DALYs) among frontier scenarios. Panels show results for (a) Democratic Republic of Congo, (b) Niger, and (c) Togo. Each point represents a cost-efficient scenario on the efficiency frontier. Blue points indicate scenarios targeting all children aged 6–59 months, whereas orange points indicate scenarios prioritizing children aged 6–23 months. DALYs are expressed in thousands.

In the DRC and Niger, approximately a quarter of the identified frontier scenarios adopt a mixed-balanced approach (Fig. 3). A balanced approach refers to scenarios in the frontier where the share of facility-based delivery and campaign-based delivery is both above 40%, meaning that no service delivery modality dominates over the other. Conversely, in Togo, scenarios on the frontier are skewed toward campaign delivery. Indeed, Togo achieves high coverage through low-cost campaigns (Table 2), shifting the efficiency frontier toward campaign prioritization.

**Table 2:** Share of scenarios prioritizing a certain service delivery modality in frontier scenarios.

	Prioritizing facility			Prioritizing campaign		
	DRC	Niger	Togo	DRC	Niger	Togo
All frontier scenarios	17%	15%	0%	51%	76%	100%
Across urban populations	51%	41%	0%	42%	42%	100%
Across rural populations	12%	19%	0%	85%	75%	100%
Across children 6–23 months	17%	15%	0%	51%	76%	100%
Across children 24–59 months	48%	49%	35%	29%	25%	43%

The table reports the proportion of frontier scenarios that prioritize facility-based or campaign-based delivery of vitamin A supplementation, overall and across population subgroups, including urban and rural settings and age groups, for each study country.

Analyzing urban and rural settings separately revealed distinct patterns in VAS delivery (Table 2). For rural populations, campaign service delivery is consistently prioritized across all frontier scenarios in all three countries. For urban populations, facility-based delivery is prioritized in frontier scenarios for both DRC and Niger. In contrast, in Togo, campaigns dominate for both rural and urban settings.

The total cost of VAS frontier scenarios varied significantly across the countries studied (Table 4). The DRC exhibited the highest average costs for frontier scenarios (average USD 5.48 million, with variability up to USD 9.74 million), whereas Niger maintained lower costs (average of USD 1.66 million), and Togo achieved the most cost-efficiency with an average cost of frontier scenarios of only USD 116 241.

Scenarios where campaign-based delivery is the predominant delivery platform are consistently more resource-intensive than frontier scenarios prioritizing facility-based delivery (Table 3). In both the DRC and Niger, campaigns cost just over twice as much as facility-based delivery for all frontier scenarios (2.1 times in DRC, 2.09 times in Niger). The disparity is relevant in rural areas, where VAS delivery is nearly three times more expensive (2.81 times in the DRC, 2.66 times in Niger) due to the predominance of campaign-based delivery assigned to children in rural settings in the frontier scenarios.

**Table 3:** Cost ratio of campaign-based to facility-based vitamin A supplementation scenarios.

Scenarios	DRC	Niger	Togo
All frontier scenarios	2.1	2.09	NA
Across urban populations	1.81	1.34	NA
Across rural populations	2.81	2.66	NA
Across children 6–23 months	3.15	3.25	NA
Across children 24–59 months	1.79	1.82	1.06

The table presents the ratio of average program costs for campaign-based delivery relative to facility-based delivery across frontier scenarios, reported overall and by population subgroup for each country. Values greater than one indicate higher costs for campaign-based delivery.

### Incremental cost-effectiveness relative to current national strategies

**Figure 4:**
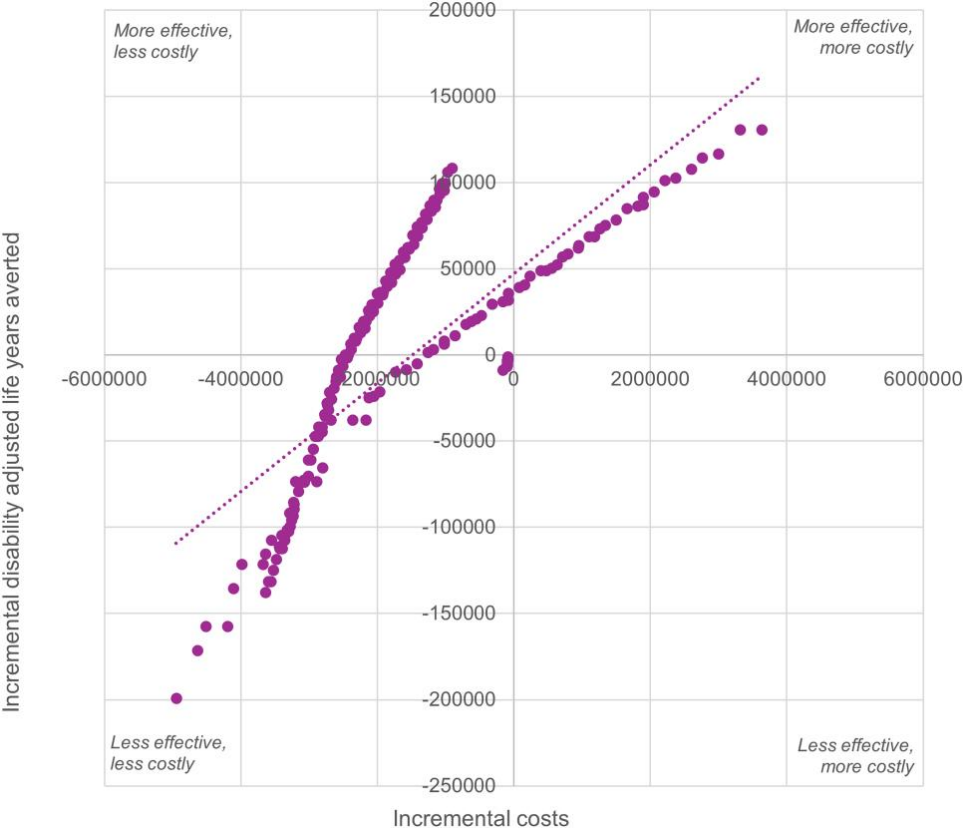
Incremental costs and incremental disability-adjusted life years (DALYs) averted across frontier scenarios. The figure presents incremental costs plotted against incremental DALYs averted for frontier scenarios relative to the baseline. Each point represents a simulated cost-efficient scenario, illustrating the trade-off between additional program costs and health gains achieved through alternative vitamin A supplementation delivery and targeting strategies.

Incremental cost-effectiveness analysis compared frontier scenarios to each country’s current national VAS delivery strategy. As illustrated in Fig. 4, a positive, nonlinear relationship is observed between incremental costs and DALYs averted. As investment increased, health outcomes improved. However, the rate of health gain declined at higher cost levels.

Country-level results revealed distinct patterns in the categorization of frontier scenarios based on their ICER relative to baseline (Table 4). In the DRC, scenarios were evenly split: 40% were classified as “Less Effective but Less Costly,” yielding average savings of approximately USD 3.1 million but resulting in 83 603 additional DALYs; 20% were “More Effective and Less Costly,” with an average savings of USD 643 000 and 18 573 DALYs averted; and 40% were “More Effective but More Costly,” requiring an average additional USD 1.48 million to avert 78 023 DALYs. In Niger, 55% of scenarios were “More Effective and Less Costly,” offering average savings of USD 1.7 million and averting 51 357 DALYs, whereas the remaining 45% were “Less Effective but Less Costly,” saving USD 3 million on average but with 64 244 DALYs lost. No scenarios in Niger fell into the “More Effective but More Costly” category. In Togo, all scenarios were “Less Effective but Less Costly,” with modest average savings of USD 95 759 and 3963 DALYs lost. The average ICER for Togo is $30 per DALY averted (“Less Effective but Less Costly”), between −$62 (“More Effective and Less Costly”) and $77 per DALY averted (“Less Effective but Less Costly”) in Niger, and between −$70 (“​​More Effective and Less Costly”) and $67 per DALY averted (“Less Effective but Less Costly”) in the DRC (Table 5). Moreover, in all countries, over 95% of frontier scenarios either had ICERs below the threshold of one GDP per capita per DALY averted or resulted in negative ICERs due to cost savings and more effectiveness (Robinson et al. 2017).

**Table 4:** Average difference in DALYs and costs among frontier scenarios relative to baseline by incremental cost-effectiveness categories and country.

Country/Increment	Less effective but less costly^a^	More effective and less costly^b^	More effective but more costly^c^
***DRC***			
Incremental DALYs (average)	−83 603	18 573	78 023
Incremental costs (average)	−3 092 483	−643 057	1 483 451
*Niger*			
Incremental DALYs (average)	−64 244	51 357	NA
Incremental costs (average)	−3 019 701	−1 716 180	NA
***Togo***			
Incremental DALYs (average)	−3963	NA	NA
Incremental costs (average)	−95 759	NA	NA

^a^Based on 24 frontier scenarios for DRC, 45 frontier scenarios for Niger, and 23 frontier scenarios for Togo

^b^Based on 13 frontier scenarios for DRC and 54 frontier scenarios for Niger

^c^Based on 28 frontier scenarios for DRC

The table reports the average incremental DALYs and incremental costs for frontier scenarios classified as less effective but less costly, more effective and less costly, and more effective but more costly, relative to the baseline scenario, for each country.

**Table 5:** Average ICER (USD/DALY) observed by scenario cost-effectiveness category and country.

Country	Less effective but less costly	More effective and less costly	More effective but more costly
**DRC**			
Average ICER	67	−70	17
Min ICER	25	−350	2
Max ICER	287	−2	28
**Niger**			
Average ICER	77	−62	
Min ICER	26	−366	
Max ICER	402	−8	
**Togo**			
Average ICER	30		
Min ICER	15		
Max ICER	89		

The table summarizes the average, minimum, and maximum ICERs observed among frontier scenarios, disaggregated by cost-effectiveness category and country.

Scenarios with low ICERs (defined as those with ICERs lower than USD 15 per DALY averted) displayed distinct delivery modality characteristics (Table 6). In the DRC, these scenarios typically assigned 61% of children to campaigns and 39% to facilities, with campaigns more pronounced in rural areas (76%) and a more balanced approach in urban areas (54% facility and 46% campaign). In Niger, campaigns dominated, accounting for 81% of children reached (98% in rural and 64% in urban settings). Togo’s single low-ICER scenario showed a balanced 50/50 split between facilities and campaigns across both urban and rural settings. Coverage among children aged 6–23 months was consistently high across all three countries (93%–95%) in these low-ICER scenarios. However, achieved coverage for children aged 24–59 months varied significantly: 55% in the DRC, 89% in Niger, and 25% in Togo.

**Table 6:** Key characteristics of scenarios with ICER lower than USD 15 per DALY averted.

Scenario characteristics	DRC	Niger	Togo
**Number of scenarios**	**15**	**13**	**1**
**Modality used to delivery VAS**			
Share of children assigned to facility	39%	19%	50%
Share of children assigned to campaign	61%	81%	50%
***Among urban settings***			
Share of children assigned to facility	54%	36%	50%
Share of children assigned to campaign	46%	64%	50%
***Among rural settings***			
Share of children assigned to facility	24%	2%	50%
Share of children assigned to campaign	76%	98%	50%
**Coverage achieved**			
Among children 6–23 months	93%	93%	95%
Among children 24–59 months	55%	89%	25%

The table describes the delivery modality mix, population coverage patterns, and achieved coverage levels among frontier scenarios with ICERs below USD 15 per DALY averted, reported separately for each country.

On average, scenarios on the efficiency frontier consistently resulted in cost savings relative to the baseline across all countries. Specifically, the average total costs for frontier scenarios were 60% lower than baselines in Niger and 48% lower in Togo, whereas in the DRC, they were 16% lower.

### Sensitivity analysis

Sensitivity analyses examined the robustness of base-case findings to changes in key delivery conditions identified through stakeholder engagement. Three structural variations were assessed: (i) constraining realized facility-based coverage by capsule availability, (ii) introducing delivery through ECE platforms for older children based on country-specific ECE coverage, and (iii) strengthening facility-based delivery capacity by increasing the probability of receiving VAS conditional on assignment.

Supply constraints affecting facility-based delivery substantially altered the efficiency frontier in the DRC and Niger, displacing a large share of base-case frontier scenarios and reducing achievable coverage. In the DRC, only one-third of the original cost-efficient scenarios remained on the efficiency frontier, whereas in Niger, approximately half were displaced. Togo was minimally affected, as most of its cost-efficient scenarios relied on campaign-based delivery rather than facilities (Table 7). This shift indicates that strategies considered efficient under normal conditions may lose their cost-effectiveness when supply chains are unreliable.

**Table 7:** Share of frontier scenarios that remain in the frontier after sensitivity analysis.

Country	Supply in facilities	Integration into ECE	Facility strengthening
DRC	29%	89%	48%
Niger	45%	53%	23%
Togo	100%	9%	9%

The table reports the proportion of original frontier scenarios that remain cost-efficient after applying alternative assumptions related to facility supply constraints, integration into early childhood education, and facility capacity strengthening, by country.

In contrast, the introduction of ECE-based delivery had a moderate effect, primarily influencing strategies targeting children aged 36–59 months in countries with higher ECE enrollment rates (Table 7). In the DRC, where ECE enrollment is below 10%, approximately 90% of previously efficient scenarios remained on the frontier, indicating that integration had little effect on which strategies were most cost-effective. In contrast, in Niger—where enrollment levels are higher—only about half of the original frontier scenarios remained efficient. This suggests that ECE integration can significantly alter the relative cost-effectiveness of delivery options in settings with higher enrollment, potentially by shifting delivery modalities or increasing logistical complexity.

Strengthening facility-based delivery (defined as increasing coverage probability to 80% for children under 2 years and by 50% for children aged 2–5 years) produced the largest gains in coverage and health impact, shifting the efficiency frontier upward and reducing reliance on campaign-based delivery. It displaced approximately 50% of frontier scenarios in the DRC, 75% in Niger, and nearly 90% in Togo. This shift was accompanied by a decrease in the share of children reached through campaigns, reflecting a pivot toward facility-based delivery. Ultimately, strengthened facility delivery resulted in a more productive frontier (Table 8).

**Table 8:** Changes in average coverage achieved, cost, and cost per percentage point of coverage achieved after sensitivity analysis by Country^a^

Country	Average coverage achieved	Total cost (USD)	Cost per percentage point coverage achieved
*Original frontier*			
DRC	68%	5 938 228	87 734
Niger	64%	1 856 451	29 193
Togo	75%	119 028	1577
*Supply constraint*			
DRC	58%	5 567 370	95 633
Niger	54%	1 632 101	30 224
Togo	72%	104 558	1459
*ECE integration*			
DRC	70%	6 155 132	88 101
Niger	63%	1 828 680	29 032
Togo	90%	118 794	1320
*Strengthening Facility capacity to deliver integrated VAS*			
DRC	74%	5 888 911	79 318
Niger	73%	1 936 621	26 562
Togo	95%	125 393	1320

The table compares the average coverage levels, total program costs, and cost per percentage point of coverage achieved across the original frontier and alternative sensitivity analysis scenarios. The results focus on scenarios targeting all children aged 6–59 months.

^a^The analysis focuses on scenarios targeting all children aged 6–59 months. Scenarios that exclusively targeted children under 2 years were excluded to improve comparability in cost and coverage results. This exclusion accounts for the fact that interventions like ECE primarily impact children older than 2 years.

Overall, sensitivity analyses highlight that the relative cost-effectiveness of delivery strategies is highly contingent on system readiness, particularly supply reliability and facility performance, underscoring the importance of addressing these constraints when considering transitions away from campaign-based delivery.

## Discussion

### Contextual variation and the case for country-specific strategies

This study provides compelling evidence that the cost-effectiveness of VAS delivery is highly context-dependent, reinforcing the need for locally tailored strategies to maximize public health impact. The identification of markedly different cost-efficient scenarios across the DRC, Niger, and Togo illustrates how operational realities, health system capacities, and cost structures shape optimal delivery approaches. These variations highlight that no single delivery modality or strategy can be universally applied across settings with equal effectiveness.

### Prioritizing children aged 6–23 months

Across all countries, the prioritization of children aged 6–23 is consistently among the most cost-effective scenarios. This age group yields the highest marginal health gains, as reflected by steeper reductions in DALYs, particularly in the DRC and Niger, underscoring the value of targeting younger children to maximize the impact of VAS investments. Children aged 6–23 months are particularly vulnerable. Approximately 75% of under-5 mortality in West and Central Africa occurs within the first 2 years of life (Laillou et al. 2024). Crucially, VAS leads to a significantly higher mean reduction in deaths for children aged 6–23 months (7.1 deaths per 1000 live births) compared with older children aged 24–59 months (2.5 deaths per 1000 live births) (Laillou et al. 2024). Furthermore, from an economic perspective, the mean cost per averted child death was found to be 2.8 times lower for the 6- to 23-month age group compared with the 24- to 59-month age group across 20 countries in the region (Laillou et al. 2024).

### Assessing the efficiency of delivery modalities

Although campaigns generally achieve high coverage, they are costly. In the DRC and Niger, campaigns, although effective in reaching children, are substantially more costly than routine delivery. The total cost of campaigns is roughly double that of facility-based delivery in the DRC and Niger. Evidence from Senegal shows that campaigns achieve higher coverage than routine delivery but at substantially higher costs (Horton et al. 2018). Moreover, the baseline cost per child covered for campaigns in the DRC (USD 0.55) and Niger (USD 0.56) is higher than for routine distribution (USD 0.24 in the DRC and USD 0.30 in Niger). In Senegal, it is estimated that campaigns cost USD 1.25 per child per year for VAS (Horton et al. 2018). Moreover, the results from the frontier analysis show that campaign delivery should be consistently prioritized for rural populations across all frontier scenarios in the DRC and Niger. This emphasizes the effectiveness of campaigns for reaching marginalized or remote populations.

On the other hand, routine delivery can achieve high coverage at lower costs—particularly in the DRC and Niger. The frontier analysis shows that facility-based delivery is prioritized in frontier scenarios for both the DRC and Niger across urban populations. Routine delivery, although sometimes preferred for its perceived hygiene and professional oversight, faces challenges in reaching older children (over 12 months) and those in remote areas of Senegal. Its effectiveness relies heavily on robust record-keeping and strong community support (Horton et al. 2018). Research in Ethiopia comparing Child Health Days with routine delivery notes that campaigns offer advantages in ease of mobilization and reaching children in remote areas, although routine delivery ensures coverage for those who might miss campaign days and avoids disrupting other essential health services (Horton et al. 2018).

These trade-offs between high-coverage and high-cost of campaigns in the DRC and Niger have interesting implications in the efficiency frontier analysis. In the DRC and Niger, approximately a quarter of the identified frontier scenarios adopt a balanced approach between campaign and routine. This suggests that the most efficient VAS in Niger and the DRC can be achieved through adopting both service delivery modalities, depending on geographic location.

However, Togo achieves campaign coverage at a much lower cost, just USD 0.10 per child, where integration with CHW programs improves efficiency. Indeed, scenarios on the frontier for Togo are more skewed toward campaign delivery. This observation aligns with Togo's high coverage achieved through mass outreach activities and its significantly lower costs compared with facility-based delivery, which shifts the efficiency frontier toward campaign prioritization. Similarly, VAS in Nigeria can be cost-efficiently delivered through campaigns when integrated with Seasonal Malaria Chemoprevention (SMC) campaigns. In this integrated approach, the additional cost of delivering VAS can be modest (USD 0.24 per child in one study) (Oresanya et al. 2024) and approximately USD 0.45 per child reached in another (GiveWell 2024). This cost-efficiency is achieved by leveraging an existing service delivery modality, thereby distributing the fixed costs across multiple interventions (GiveWell 2024). This integrated model is cost-effective, with estimates ranging from USD 760 to USD 880 per death averted (GiveWell 2024).

This difference between countries further reinforces the importance of adapting strategies to local operational realities. The continued effectiveness of campaigns in reaching high coverage among vulnerable groups highlights the enduring need for such modalities in contexts where routine systems remain weak.

### Comparative cost and cost-effectiveness of VAS delivery

Overall, VAS is highly cost-effective in all three countries. Using the WHO threshold, over 95% of its cost-effective scenarios had ICERs below the threshold of one GDP per capita per DALY averted.

Given the unique scope of this study, comparisons are challenging. However, a study in Ethiopia found similar levels of cost-effectiveness for their scale-up of campaigns. VAS through campaigns saved 20 200 lives and averted 230 000 DALYs of children 6–59 months. The cost per DALY averted was equivalent to 6% of per capita GDP (USD 9), thus cost-effective according to the WHO criteria (Fiedler and Chuko 2008). On the other hand, the results from a microsimulation study in Nigeria, Kenya, and Burkina Faso suggest that VAS may no longer be as cost-effective as it has been previously. The study estimated the cost and effect of scaling up VAS, with ICERs of USD 860/DALY in Nigeria, USD 550/DALY in Kenya, and USD 220/DALY in Burkina Faso.

This highlights the principle that the cost-effectiveness of health interventions is not static but profoundly context-dependent, aligning with the attached study’s own conclusions. Although VAS remains highly cost-effective in the specific contexts of the DRC, Niger, and Togo, literature has not found that VAS is consistently cost-effective.

### Resource intensity of campaigns

A key finding of this study is that while campaigns offer significant scale and reach for VAS delivery, particularly in rural areas, their resource intensity raises concerns about long-term financial sustainability. Campaigns are often heavily reliant on donor funding, which can be unpredictable (Eldridge and Keller 2025). This reliance on external funding can hinder the development of sustainable, government-owned programs, especially in resource-constrained low-and middle-income countries (Ahmed et al. 2025). For example, a study in Senegal highlighted the difficulty the government faced in absorbing campaign costs initially covered by partner organizations (Horton et al. 2018). Addressing the sustainability of VAS campaigns, therefore, requires optimizing delivery and broader reforms in nutrition financing and health system strengthening. This includes reducing reliance on short-term, vertical funding and fostering national ownership and capacity to absorb intervention costs.

### Strengthening routine systems for sustainable coverage

Insights from the sensitivity analysis are vital for building resilient VAS programs. Disruptions to facility supply chains were shown to have the most detrimental effect on cost-efficiency and productivity, emphasizing that robust supply systems are not merely logistical details but fundamental determinants of program success and sustainability. As a result, strengthening facility-based delivery can improve the long-term sustainability of VAS.

This insight is strongly supported by existing literature on health system strengthening. Integrated strategies are considered more sustainable and effective for improving maternal and under-5 nutrition, health, and immunization services (Khan et al. 2025). Examples of health system strengthening interventions directly relevant to VAS delivery include improving supply chain management to reduce stock-outs, strengthening community health worker (CHW) programs, and enhancing referral systems (Kudymowa et al. 2025).

Insights from this study thus suggest that investment in health systems strengthening, such as improved facility reach and robust supply chains, translates into increased effectiveness and productivity of routine VAS delivery. This, in turn, fosters enhanced long-term sustainable coverage and reduces the reliance on potentially more costly campaign-based approaches. Although campaigns may be necessary for immediate reach, a strategic long-term vision for VAS delivery must involve significant, realistic budgeting for upfront investments in routine health system infrastructure, human resources, and supply chains to ensure sustainable access to essential services.

### Limitations

Despite these valuable insights, the study acknowledges several limitations that warrant consideration for the interpretation and comparability of results. First, significant discrepancies were observed in coverage estimates across different data sources. Relying on observed estimates from country offices for the baseline, although the decision model used household budget surveys and global data sources, introduced inconsistencies that primarily impact the interpretation of service effectiveness and influence cost estimates, especially those derived from top-down expenditure approaches. Second, the reliance on budget estimates rather than actual expenditure data introduces potential inaccuracies, as budgets may not fully reflect actual spending patterns or resource allocations. Third, costs are estimated at the program level and may overstate the strictly incremental cost of adding VAS to existing service contacts; however, this perspective is appropriate for comparing delivery strategies under routine implementation conditions. Finally, there were insufficient data available to conduct subnational analyses. These limitations underscore the imperative for improved data collection and harmonization of coverage measurement methodologies across countries to enhance the certainty and comparability of future analyses.

### Future research

Future research should explore the long-term sustainability and scalability of cost-effective VAS delivery strategies, particularly given varying national capacities. Additionally, it would be interesting to conduct subnational analyses. A central recommendation is the harmonization of coverage measurement and strengthening financial data monitoring across data sources. Further investigation into the factors contributing to varying country capacities for consistent coverage is also warranted.

## Conclusion

This study provides comparative evidence on the cost-effectiveness of VAS delivery strategies in the DRC, Niger, and Togo, highlighting how delivery modality, system capacity, and context-specific constraints shape program efficiency and impact. Although all three countries can achieve cost-efficient outcomes, the pathways differ: campaigns remain essential for reaching underserved populations but are significantly more costly in some settings, whereas routine delivery offers more sustainable potential when adequately supported. The findings highlight the importance of prioritizing younger children, preventing supply shortages, and tailoring delivery models to local health system realities. Moving forward, improved harmonization of coverage measurement and financial tracking will be critical for enabling more accurate assessments and better-informed resource allocation. Ultimately, country-specific strategies that reflect both efficiency and equity considerations will be key to sustaining and scaling effective VAS programs.

## Contributorship statement

Conception or design of the work: Dr Arnaud Laillou, Dr Andreas Hasman, and Natsuki Kawai. Data collection: Dr Lucia Corball, Hannah Rowett, and Kenya Chappel. Data analysis and interpretation: Dr Lucia Corball, Hannah Rowett, and Kenya Chappel. Drafting the article: Dr Lucia Corball, Hannah Rowett, and Kenya Chappel. Critical revision of the article for important intellectual content: Dr Andreas Hasman, Dr Arnaud Laillou, Natsuki Kawai, Dr Lucia Corball, Hannah Rowett, and Kenya Chappel. Final approval of the version to be submitted: All authors reviewed and approved the final manuscript prior to submission and agree to be accountable for all aspects of the work.

## Supplementary Material

czag032_Supplementary_Data

## Data Availability

The data underlying this article are available in the article and in its online supplementary material.

## Reflexivity statement

The author group brings together a diverse mix of disciplinary backgrounds, seniority levels, and institutional perspectives. The team includes four female and two male authors and comprises three junior and three senior researchers. Four authors specialize in public health nutrition, including one with specific expertise in vitamin A supplementation, all with experience in nutrition policy and program and operational implementation. The remaining two authors specialize in public health and health economics and public financial management, respectively. Authors are based across multiple geographic locations, including West and Central Africa, Southern Africa, Europe, and North America, and include researchers working within international organizations as well as those based in consulting and research institutions. All authors have experience working with governments and partners in low- and middle-income countries, most notably through direct engagement with national health and nutrition systems in West and Central Africa. This diversity of perspectives informed the framing of the research questions, interpretation of results, and consideration of policy relevance, while the authors recognize the continued importance of strengthening representation of researchers based in study countries in future work.
