# Supplementary material for: Cost-effectiveness analysis of vitamin A supplementation delivery modalities in the DRC, Togo, and Niger: informing sustainable program design
Source: Health Policy Plan. 2026 Mar 11;41(5):863–76. doi: 10.1093/heapol/czag032 (PMC13187644; doi:10.1093/heapol/czag032)
Supplement: czag032_Supplementary_Data [file czag032_supplementary_data.zip › Supplementary Material 1_CHEERS-2022-checklist.docx]

CHEERS 2022 Checklist

|  | **Item** | **Guidance for Reporting** | **Reported in section** |
| --- | --- | --- | --- |
| **TITLE** | | |  |
| Title | 1 | Identify the study as an economic evaluation and specify the interventions being compared. | Title (“Cost-Effectiveness Analysis of Vitamin A Supplementation Delivery Modalities…”) |
| **ABSTRACT** | | |  |
| Abstract | 2 | Provide a structured summary that highlights context, key methods, results and alternative analyses. | Abtract |
| **INTRODUCTION** | | |  |
| Background and objectives | 3 | Give the context for the study, the study question and its practical relevance for decision making in policy or practice. | Introduction, paras 1–8 (from VAD burden through research questions) |
| **METHODS** | | |  |
| Health economic  analysis plan | 4 | Indicate whether a health economic analysis plan was developed and  where available. | Not applicable |
| Study population | 5 | Describe characteristics of the study population (such as age range, demographics, socioeconomic, or clinical characteristics). | Methods 2.2 (children 6–59 months; age and urban/rural stratification) |
| Setting and location | 6 | Provide relevant contextual information that may influence findings. | Introduction (country context); Methods 2.1–2.2 |
| Comparators | 7 | Describe the interventions or strategies being compared and why chosen. | Introduction (delivery modalities); Methods 2.1, 2.3 |
| Perspective | 8 | State the perspective(s) adopted by the study and why chosen. | Methods 2.5 (health service provider perspective) |
| Time horizon | 9 | State the time horizon for the study and why appropriate. | Methods 2.2 (1-year costing; lifetime health outcomes) |
| Discount rate | 10 | Report the discount rate(s) and reason chosen. | Methods 2.2 (3% annual discounting) |
| Selection of outcomes | 11 | Describe what outcomes were used as the measure(s) of benefit(s) and harm(s). | Methods 2.1, 2.3 (DALYs averted) |
| Measurement of outcomes | 12 | Describe how outcomes used to capture benefit(s) and harm(s) were measured. | Methods 2.3 (Mortality and morbidity effects) |
| Valuation of outcomes | 13 | Describe the population and methods used to measure and value outcomes. | Methods 2.3 |
| Measurement and valuation of resources  and costs | 14 | Describe how costs were valued. | Methods 2.5 (bottom-up and top-down costing; cost categories) |
| Currency, price date, and conversion | 15 | Report the dates of the estimated resource quantities and unit costs, plus the currency and year of conversion. | Methods 2.5 + Supplementary Appendix S2 |
| Rationale and  description of model | 16 | If modelling is used, describe in detail and why used. Report if the model  is publicly available and where it can be accessed. | Methods 2.1 & Figure 1 (decision tree; scenario-based optimisation) |
| Analytics and assumptions | 17 | Describe any methods for analysing or statistically transforming data, any extrapolation methods, and approaches for validating any model used. | Methods 2.1, 2.3, 2.6 |
| Characterizing heterogeneity | 18 | Describe any methods used for estimating how the results of the study vary for sub-groups. | Methods 2.2, 2.3 (age group; urban/rural); Results 3.1–3.2 |
| Characterizing  distributional effects | 19 | Describe how impacts are distributed across different individuals  or adjustments made to reflect priority populations. | Not applicable |
| Characterizing uncertainty | 20 | Describe methods to characterize any sources of uncertainty in the analysis. | Methods 2.7 (scenario-based sensitivity analyses) |
| Approach to engagement with patients and others affected by the study | 21 | Describe any approaches to engage patients or service recipients, the general public, communities, or stakeholders (e.g., clinicians or payers) in the design of the study. | Methods 2.7 (key informant input informing sensitivity scenarios) |
| **RESULTS** | | |  |
| Study parameters | 22 | Report all analytic inputs (e.g., values, ranges, references) including uncertainty or distributional assumptions. | Results 3.1–3.2; Tables 4–9 |
| Summary of main results | 23 | Report the mean values for the main categories of costs and outcomes of interest and summarise them in the most appropriate overall measure. | Results 3.1–3.2; Tables 4–9 |
| Effect of uncertainty | 24 | Describe how uncertainty about analytic judgments, inputs, or projections  affect findings. Report the effect of choice of discount rate and time horizon, if applicable. | Results 3.3.4; Tables 10–11 |
| Effect of engagement with patients and others affected by the study | 25 | Report on any difference patient/service recipient, general public, community, or stakeholder involvement made to the approach or findings of the study | Results 3.3.4 |
| **DISCUSSION** | | |  |
| Study findings, limitations, generalizability, and current knowledge | 26 | Report key findings, limitations, ethical or equity considerations not captured, and how these could impact patients, policy, or practice. | Discussion (all subsections incl. Limitations) |
| **OTHER RELEVANT INFORMATION** | | | |
| Source of funding | 27 | Describe how the study was funded and any role of the funder in the identification, design, conduct, and reporting of the analysis | End matter / Acknowledgements |
| Conflicts of interest | 28 | Report authors conflicts of interest according to journal or  International Committee of Medical Journal Editors requirements. | End matter |

Husereau D, Drummond M, Augustovski F, de Bekker-Grob E, Briggs AH, Carswell C, Caulley L, Chaiyakunapruk N, Greenberg D, Loder E, Mauskopf J, Mullins CD, Petrou S, Pwu RF, Staniszewska S; CHEERS 2022 ISPOR Good Research Practices Task Force. Consolidated Health Economic Evaluation Reporting Standards 2022 (CHEERS 2022) Statement: Updated Reporting Guidance for Health Economic Evaluations. BMJ. 2022;376:e067975.

The checklist is Open Access distributed in accordance with the terms of the Creative Commons Attribution (CC BY 4.0) license, which permits others to distribute, remix, adapt and build upon this work, for commercial use, provided the original work is properly cited. See: [http://creativecommons.org/licenses/by/4.0/.](http://creativecommons.org/licenses/by/4.0/)
