# Supplementary material for: Cost-effectiveness analysis of vitamin A supplementation delivery modalities in the DRC, Togo, and Niger: informing sustainable program design
Source: Health Policy Plan. 2026 Mar 11;41(5):863–76. doi: 10.1093/heapol/czag032 (PMC13187644; doi:10.1093/heapol/czag032)
Supplement: czag032_Supplementary_Data [file czag032_supplementary_data.zip › Supplementary Material 2.docx]

### Annex A: Data Sources for Model Parameters used in the Economic Analysis

| **Model parameters** | **Data sources** |
| --- | --- |
| **Probabilities used in the decision model** | |
| Probability of the beneficiary being between 6-23 months vs. 24-59 months | DHS surveys (DRC 2014, Togo 2014, Niger 2017) |
| Probability of the beneficiary living in an urban setting vs. rural setting | United Nations, Department of Economic and Social Affairs, Population Division (2018). World Urbanization Prospects: The 2018 Revision, Online Edition. |
| Probability of the beneficiary of being assigned to facility vs. campaign service delivery | This was modelled and iterated across all scenarios generated |
| Probability of the beneficiary receiving VAS under the service delivery modality assigned vs. not receiving | Estimates of effective coverage by service delivery modality from campaign/outreach data, DHIS data and key informant interviews with country government officials and UNICEF Country Offices Nutrition Specialists |
| Probability of diarrhea among children receiving VAS | Reduction of overall baseline probability from A. Imdad, E. Mayo-Wilson, M.R. Haykal et al. (2022). ‘Vitamin A supplementation for preventing morbidity and mortality in children from six months to five years of age’, Cochrane Database of Systematic Reviews 2022, 3. |
| Probability of diarrhoea among children not receiving VAS | Institute for Health Metrics and Evaluation (IHME). GBD Results. Seattle, WA: IHME, University of Washington, 2024. Available from https://vizhub.healthdata.org/gbd-results/(link is external). (Accessed 10 August 2024) |
| Probability of measles among children receiving VAS | Reduction of overall baseline probability from A. Imdad, E. Mayo-Wilson, M.R. Haykal et al. (2022). ‘Vitamin A supplementation for preventing morbidity and mortality in children from six months to five years of age’, Cochrane Database of Systematic Reviews 2022, 3. |
| Probability of measles children not receiving VAS | Institute for Health Metrics and Evaluation (IHME). GBD Results. Seattle, WA: IHME, University of Washington, 2024. Available from https://vizhub.healthdata.org/gbd-results/(link is external). (Accessed 10 August 2024) |
| Probability of stunting due to diarrhea, by stunting severity | Checkley W, Buckley G, Gilman RH, et al. Multi-country analysis of the effects of diarrhoea on childhood stunting. Int J Epidemiol 2008. |
| Probability of death due to diarrhoea among children receiving VAS | Reduction of overall baseline probability from A. Imdad, E. Mayo-Wilson, M.R. Haykal et al. (2022). ‘Vitamin A supplementation for preventing morbidity and mortality in children from six months to five years of age’, Cochrane Database of Systematic Reviews 2022, 3. |
| Probability of death due to diarrhoea among children not receiving VAS | Institute for Health Metrics and Evaluation (IHME). GBD Results. Seattle, WA: IHME, University of Washington, 2024. Available from https://vizhub.healthdata.org/gbd-results/(link is external). (Accessed 10 August 2024) |
| Probability of death adjusted by stunting severity and cause of death | Olofin I, McDonald CM, Ezzati M, et al. Associations of Suboptimal Growth with All-Cause and Cause-Specific Mortality in Children under Five Years: A Pooled Analysis of Ten Prospective Studies. PLOS One 2013; 8(5): e64636. |
| Probability of death due to measles | Institute for Health Metrics and Evaluation (IHME). GBD Results. Seattle, WA: IHME, University of Washington, 2024. Available from https://vizhub.healthdata.org/gbd-results/(link is external). (Accessed 10 August 2024) |
| **Costs used in the decision model** | |
| VAS service delivery costs | Expenditure data for mass campaigns and outreach activities in each country for 1 year (2023) broken down by input and cost categories. For facility-based delivery, data on labour costs were obtained from government officials and UNICEF country offices. Other program costs (such as program management, etc) were modelled based on assumptions based on WHO-CHOICE estimates and key informant interviews with nutrition officers. |
| Vitamin A capsules costs | UNICEF Country Offices procurement data |

### Annex B: Components for the Estimation of Children Receiving VAS

| Component | Definition | VAS-specific | Note on modelling |
| --- | --- | --- | --- |
| Target population | The age-determined population that could possibly receive the intervention | Children between 6 and 23 months  Children between 24 and 59 months | Based on country-specific population estimates shared by country officers and/or UNICEF or HKI officers. |
| Population in need | Share of the target population that requires the intervention per year | 100% of the target population. The intervention is preventative and universal, therefore, the population in need of the intervention is the entire target population. | Assumed to remain at 100%. There is no evidence to support a shift in the population in need of VAS. |
| Coverage | Share of the population in need who is expected to receive VAS by service delivery modality | Campaign/mass outreach activities: 90% for all countries    Facility-based delivery  Liberiaᵃ:7% - DHS  Togo:90%- DHS  DRC:91%- DHS  Niger: 88% - DHS | Overall coverage will be optimised in relation to costs through scenario analysis. Scenarios are described later in this document.  We assumed a probability of coverage through campaigns/mass outreach activities of 90%. The probability of coverage through facility-based delivery took the same value of coverage reported in the latest DHS survey and was adjusted to account for urban/rural differences with respect to the average. |

###

### Annex C: Key model parameters by country

**Table C-1: Key model parameters by country**

| **Parameter category** | **Parameter** | **Age group** | **Urban/Rural** | **DRC** | **Niger** | **Togo** | **Source** |
| --- | --- | --- | --- | --- | --- | --- | --- |
| Coverage (%) | Probability of coverage – facility | 6–23 | Urban | 81.2 | 88.0 | 75.8 | DHS / Country MOH |
| Coverage (%) | Probability of coverage – facility | 6–23 | Rural | 60.9 | 72.0 | 73.5 | DHS / Country MOH |
| Coverage (%) | Probability of coverage – facility | 24–59 | Urban | 21.6 | 33.0 | 37.9 | DHS / Country MOH |
| Coverage (%) | Probability of coverage – facility | 24–59 | Rural | 16.2 | 27.0 | 36.8 | DHS / Country MOH |
| Coverage (%) | Probability of coverage – campaign | 6–23 | All | 95% | 95% | 95% | DHS / KI |
| Coverage (%) | Probability of coverage – campaign | 24–59 | All | 95% | 95% | 95% | DHS / KI |
| Epidemiology | Diarrhoea incidence | 6–23 | — | 0.87 | 1.07 | 0.92 | GBD |
| Epidemiology | Diarrhoea incidence | 24–59 | — | 0.59 | 0.74 | 0.65 | GBD |
| Epidemiology | Measles incidence | 6–23 |  | 0.01 | 0.05 | 0.04 | GBD |
| Epidemiology | Measles incidence | 24–59 | — | 0.004 | 0.01 | 0.01 | GBD |
| Impact | Diarrhoea incidence RR reduction | All | — | 0.15 | | | Imdad et al. |
| Impact | Measles incidence reduction | All |  | 0.50 | | | Imdad et al. |
| Impact | Diarrhoea RR mortality reduction | All |  | 0.12 | | | Imdad et al. |
| Demography | Life expectancy at birth | — | — | 61.6 | 60.0 | 63.9 | UN |
| YLD/YLL ratio | Diarrhea | 6–23 | — | 0.01750 | 0.00634 | 0.00877 | GBD |
| YLD/YLL ratio | Diarrhea | 24–59 |  | 0.09775 | 0.01725 | 0.02721 | GBD |
| YLD/YLL ratio | Measles | 6–23 |  | 0.00590 | 0.00131 | 0.00321 | GBD |
| YLD/YLL ratio | Measles | 24–59 |  | 0.00569 | 0.00123 | 0.00296 | GBD |

**Annex D: Cost estimates of Vitamin A Supplementation**

Interventions inputs specific for VAS were mapped out with their level of utilisation, and unit or expenditure costs for each service delivery modality. The treatment inputs that were mapped and the cost are outlined in the table below. Country-specific cost data was collected for all cases. Capital costs were excluded from the assessment.

**Table D-1: Inputs for cost of VAS delivery, key assumptions and source of data**

| **Input** | **What is costed** | **Assumption** | **Source price** |
| --- | --- | --- | --- |
| Supplies | Vitamin A 100,000 IU | 2 per child 6-11 months | UNICEF Country Offices procurement data |
|  | Vitamin A 200,000 IU | 2 per child 12-59 months |  |
| Personnel | Staffed CHW | 5 min to deliver  Time apportioned based on  ● distance between facility and community,  ● mean of transport  ● frequency of visit  ● number of interventions delivered in same visit  ● number of children covered per visit | WHO Choice^^[[1]](#footnote-1)^^triangulated with country estimates |
|  | Staffed Nurse | 5 min to deliver out of 36 hours of service |  |
|  | Contracted CHW | 5 min to deliver 5 min to deliver out of the total (X) contracted service^^[[2]](#footnote-2)^^  Time apportioned based on  ● distance between facility and community,  ● mean of transport  ● frequency of visit  ● number and type of interventions delivered in same visit  ● number of children covered per visit |  |
| Transport | Fuel of CHW or staff visiting the community | Apportioned based on  ● number and type of interventions delivered in same visit  ● number of children covered per visit | Collected as expenditure data from agencies implementing campaign or mass outreach activities. |
|  | Rental of vehicles | Apportioned based on  ● number and type of interventions delivered concomitantly  ● number of children covered per visit | Collected as expenditure data from agencies implementing campaign or mass outreach activities. |
| Logistics and supply chain | Warehousing and distribution of supply |  | UNICEF Country Offices provided an estimate of freight and supply chain costs |
| Communication, media | Advertisement | VAS-specific advertisement or apportioned based on WHO Choice | Estimates of expenditure provided by UNICEF Country Offices or campaign/mass outreach implementation agencies |
| Training | Training material  Trainer cost  Course cost | VAS-specific training to be identified and costed. Alternatively, apportioned based on WHO Choice | Estimates of expenditure provided by UNICEF Country Offices or campaign/mass outreach implementation agencies |
| Monitoring | Surveillance systems  Surveillance processes not costed in other categories | VAS-specific surveillance system, staff time, and inputs to be identified and costed. Alternatively, apportioned based on WHO Choice | Estimates of expenditure provided by UNICEF Country Offices, government and campaign/mass outreach implementation agencies |
| Management |  | VAS-specific management or planning paid tools  Administrative costs of employed staff are apportioned based on WHO Choice | Estimates of expenditure provided by UNICEF Country Offices, government and campaign/mass outreach implementation agencies |

**Table D-2: Total cost of VAS delivery through campaigns (Intense Routinization) in the DRC**

|  | 6-11 months | 12-23 months | 24-59 months | Total | Share of total |
| --- | --- | --- | --- | --- | --- |
| **Intervention costs** | |  |  | **1,713,655** | **55%** |
| Labour Costs | 241,155.12 | 224,575.84 | 623,107.82 | 1,088,839 | 64% |
| Drug costs | 27,771 | 64,921 | 180,128 | 272,820 | 16% |
| Other recurrent costs | 77,959.96 | 72,600.25 | 201,436.56 | 351,997 | 21% |
| **Programme costs** |  |  |  | **1,242,279** | **40%** |
| Programme-specific human resources | 84,161.78 | 78,375.70 | 217,461.11 | 379,999 | 31% |
| Training | 55,376.10 | 51,569.02 | 143,083.34 | 250,028 | 20% |
| Monitoring and evaluation | 46,457.32 | 43,263.40 | 120,038.57 | 209,759 | 17% |
| Transport | 13,161.50 | 12,256.66 | 34,007.31 | 59,425 | 5% |
| Communication, media, and outreach | 4,158.99 | 3,873.06 | 10,746.18 | 18,778 | 2% |
| Advocacy | 8,318.36 | 7,746.48 | 21,493.36 | 37,558 | 3% |
| General programme management | 63,505.00 | 59,139.07 | 164,087.17 | 286,731 | 23% |
| **Supply chain costs** | |  |  | **95,487** | **3%** |
| Logistic | 15,105.98 | 14,067.45 | 39,031.54 | 68,205 | 71% |
| Wastage | 6,042.39 | 5,626.98 | 15,612.62 | 27,282 | 29% |
| Investment costs |  |  |  | 51,836 | 2% |
| Capital costs | - | - | - | - | 0% |
| Infrastructure | 11,480.62 | 10,691.34 | 29,664.17 | 51,836 | 100% |
| **Total** | **643,173** | **638,014** | **1,770,234** | **3,103,258** | **100%** |
| **Number of children covered** | **1,197,642** | **1,115,305** | **3,094,522** | **5,407,469** |  |
| **Cost per child covered** | **0.54** | **0.57** | **0.57** | **0.57** |  |

**Source: HKI Givewell reports**

**Notes/ Assumption:**

- All costs were converted to USD using the average exchange rate for 2023 of XOF 624 to USD 1.^^[[3]](#footnote-3)^^
- Cost of intensive Routinization for VAS in 2023 is based on most recent expenditure data (2021). Resource utilisation or input cost data was not available, posing some limitations to the analysis – as it relies on aggregate data.
- Labour costs were reported as “Service Delivery Costs” in activities funded by HKI and implemented by either HKI or Government
- Expenditure categories reported as “Costs contributed by Nutrition International” were considered to be costs of procurement of Vitamin A capsules.
- Other direct recurrent costs were reported by HKI separately. However, it was not possible to disentangle what components of service delivery are included. These other direct recurrent costs represented 13% of the total intervention (service delivery) costs reported by HKI to GiveWell in 2021.

**Table D-3:** **Total cost of VAS routine delivery in faciltiies in DRC**

|  | **6-11 months** | **12-23 months** | **24-59 months** | **Total** | **Share of total** |
| --- | --- | --- | --- | --- | --- |
| **Intervention costs** |  |  |  | **3,222,107** | **64%** |
| Labour Costs | 243,355 | 452,301 | 1,395,939 | 2,091,595 | 65% |
| Drug costs | 73,312 | 171,024 | 527,834 | 772,170 | 24% |
| Other recurrent costs | 74,695 | 69,414 | 214,233 | 358,342 | 11% |
| **Programme costs** |  |  |  | **265,269** | **5%** |
| Programme-specific human resources | 5,477 | 8,380 | 25,864 | 39,721 | 15% |
| Training | 3,914 | 6,927 | 21,380 | 32,221 | 12% |
| Supervision | 7,827 | 13,855 | 42,760 | 64,442 | 24% |
| Monitoring and evaluation | 7,827 | 13,855 | 42,760 | 64,442 | 24% |
| Communication, media, and outreach | 1,957 | 3,464 | 10,690 | 16,111 | 6% |
| Advocacy | 3,914 | 6,927 | 21,380 | 32,221 | 12% |
| General programme management | 1,957 | 3,464 | 10,690 | 16,111 | 6% |
| **Supply chain costs** |  |  |  | **231,651** | **5%** |
| Logistic | 18,328 | 42,756 | 131,958 | 193,043 | 83% |
| Wastage | 3,666 | 8,551 | 26,392 | 38,609 | 17% |
| Investment costs |  |  |  | 1,284,330 | 26% |
| Capital costs | 254,280 | 236,303 | 729,304 | 1,219,888 | 95% |
| Infrastructure | 7,827 | 13,855 | 42,760 | 64,442 | 5% |
| **Total** | **700,509** | **1,037,222** | **3,201,184** | **5,003,357** | **100%** |

Source: Data on resource requirements, utilisation and costs for facility-based delivery was collected from government offices (Ministry of Health, PRONAUT) with support from the UNICEF DRC country office. Input cost data was available, and resource requirements and utilisation were obtained for the main intervention inputs. Costs not directly linked to service delivery were derived from the Lives Saved Costing Tool (LiST). Logistic costs of capsules were obtained from the UNICEF Supply Unit in DRC.

**Notes and assumptions:**

- All costs were transformed to USD current dollars using the average exchange rate for 2023 of XOF 624 to USD 1.^^[[4]](#footnote-4)^^
- Labour costs were reported as “Service Delivery Costs” in activities funded by HKI and implemented by either HKI or the Government. Nurses were assumed to deliver the intervention in facilities, utilising 5 minutes per dose per child covered.
- Capsules costs were shared by UNICEF Country Office. An average cost per capsule of 100,000 IU and per capsule of 200,000 IU was estimated from the price per capsule between 2021 and 2023. Number of capsules used was estimated using coverage data.
- Other direct costs were estimated using empirical cost data derived from LiST. LiST uses standardized estimates derived from health interventions to estimate other direct recurrent costs. These costs are based on comprehensive reviews and analyses of similar health programs across different regions and countries. A cost of CDF 47 (USD 0,02) per visit (dose of vitamin A) was assumed.
- Capital costs were estimated by attributing a fixed amount per child covered through this modality. The attributed estimate relies on the capital capital costs associated with an outpatient visit assuming that in the same visit, at least one additional service or intervention is provided to the child. The amount attributed to capital costs for facility-based VAS is CDF 160 (USD 0.08) per child covered and derived from the OHT and are based on WHO CHOICE Study.

**Table D-4: Total and per child cost of VAS campaigns in Togo**

|  |  |  | |  | | | |
| --- | --- | --- | --- | --- | --- | --- | --- |
| **Cost category** | **6 to 11 months** | | **24 to 59 months** | | **Total (USD)** | **Share of Total (%)** |  |
| **Intervention costs** | **11,796** | | **76,173** | | **87,969** | **70** |  |
| Labour Costs | 7,804 | | 51,850 | | 59,654 | 67.8 |  |
| Drug costs | 3,977 | | 24,222 | | 28,199 | 32.1 |  |
| Other direct costs | 15 | | 101 | | 116 | 0.2 |  |
| **Programme costs** | **4,257** | | **28,284** | | **32,541** | **26** |  |
| Programme-specific human resources | 161 | | 1,073 | | 1,234 | 3.8 |  |
| Training | - | | - | | - | - |  |
| Supervision | 969 | | 6,435 | | 7,404 | 22.8 |  |
| Monitoring and evaluation | 195 | | 1,295 | | 1,490 | 4.6 |  |
| Infrastructure | - | | - | | - | - |  |
| Transport | 2,928 | | 19,456 | | 22,384 | 68.8 |  |
| Communication, media, and outreach | 4 | | 25 | | 29 | 0.1 |  |
| Advocacy | - | | - | | - | - |  |
| General programme management | - | | - | | - | - |  |
| **Supply chain costs** | **786** | | **5,219** | | **6,005** | **4** |  |
| Logistic | 417 | | 2,768 | | 3,185 | 53 |  |
| Wastage | 369 | | 2,451 | | 2,820 | 47 |  |
| **Investment costs** |  | |  | |  |  |  |
| Capital costs | - | | - | | - | - |  |
| **Total** |  | |  | | 126,515 |  |  |

Source: Microplans from MSHPAUS

Notes and assumptions:

- All costs were transformed to USD current dollars using the average exchange rate for 2023, which was XOF 624 to USD 1.^[[5]](#footnote-5)^
- Data is budget figures, not actual expenditure.
- No capital costs
- Labour costs include per diems for VAS distributors (Community Health Workers), the cost of per diems for supervisors, per diems for accountants, coordinators and regional nutrition focal points.
- Cost of capsules provided by UNICEF and Nutrition International
- Other direct costs included scissors and management forms
- Transport costs included fuel for supervisory activities and per diems for drivers.
- Logistics: Freight is estimated at 10% of capsule costs, based on UNICEF Togo data. Logistics from central to district levels follow the Nutrition Division budget.
- Wastage costs: 10% of estimated vitamin A capsule costs

**Table D-5: Cost of facility-based delivery in Togo**

| **Cost category** | **Facility-based** | |
| --- | --- | --- |
|  | **USD** | **%** |
| **Intervention costs** | **59,683** | **37** |
| Labour Costs | 27,450 | 46 |
| Drug costs | 6,805 | 11 |
| Other direct costs | 25,428 | 43 |
| **Programme costs** | **11,723** | **7** |
| Programme-specific human resources | 1,465 | 12.5 |
| Training | 1,465 | 12.5 |
| Supervision | 2,931 | 25 |
| Monitoring and evaluation | 2,931 | 25 |
| General programme management | 2,931 | 25 |
| **Supply chain costs** | **2,382** | **1** |
| Logistic | 1,701 | 29 |
| Wastage | 681 | 71 |
| **Investment costs** | **90,973** | **55** |
| Infrastructure | 2,931 | 3 |
| Capital costs | 86,849 | 97 |
| **Total** | **163,567** |  |

Source: UNICEF Togo country office.

Notes and assumptions:

- All costs were transformed to USD current dollars using the average exchange rate for 2023, which was XOF 624 to USD 1.^[[6]](#footnote-6)^
- Labour: Nurses deliver the intervention in facilities, covering 5 minutes per dose per child.
- Cost of capsules: UNICEF and Nutrition International
- Other direct costs were estimated using empirical cost data derived from LiST. LiST uses standardised estimates derived from health interventions to estimate other direct recurrent costs. These costs are based on comprehensive reviews and analyses of similar health programs across regions and countries. In the case of Vitamin A, a cost of CDF 56 (USD 0.09) per visit (dose) was assumed.
- Transport: Not included
- Logistic costs: 25% of estimated vitamin A capsule costs (excluding wastage)
- Wastage costs: 10% of total drug cost was estimated to be the cost of wastage due to storage and inventory management issues.
- Capital costs: Estimated using the Lives Saved Tool (LiST), which allocates capital costs to facility-based interventions such as VAS.

### Annex E: Costing Results of Vitamin A Supplementation

The costing results for VAS via different service delivery modalities reveal significant differences in distribution costs (Table D-1). Niger has the highest total distribution costs per campaign, over USD 3.8 million, followed by the DRC at nearly USD 3 million, while Togo has the lowest at USD 126,000.

**Table E-1: Baseline costs of vitamin A supplementation by service delivery modality, by country, in USD**

|  | Campaign | | | Routine | | |
| --- | --- | --- | --- | --- | --- | --- |
|  | DRC | Togo | Niger | DRC | Togo | Niger |
| Intervention costs | 1,635,230 | 87,969 | 1,391,328 | 2,620,864 | 77,983 | 144,523 |
| Programme costs | 1,242,279 | 32,541 | 2,113,395 | 217,169 | 6,239 | 19,351 |
| Supply chain costs | 48,599 | 6,005 | 66,099 | 293,630 | 2,382 | 5,316 |
| Capital cost | 51,836 | - | 10,173 | NA | NA | NA |
| Indirect cost (non-attributable) |  |  | 256,114 | NA | NA | NA |
| Total | 2,977,944 | 126,515 | 3,837,108 | 3,131,663 | 86,603 | 169,190 |

USD, United States Dollar

Source: Authors using internal documents from UNICEF DRC, UNICEF Togo, UNICEF Niger, Helen Keller International, Nutrition International, Ministry of Health, Hygiene and Prevention of Togo, Ministry of Public Health in Niger, Ministry of Health National Nutrition Program; Programme National de Nutrition (PRONANUT) DRC.

The cost of campaigns per child supplemented ranges from USD 0.56 for Niger, USD 0.55 for the DRC, to Togo with USD 0.10 per child ( **Table D-2**). When comparing routine VAS costs, the DRC has the highest absolute cost, but the cost per child covered is more comparable across countries, ranging from USD 0.24 in DRC to USD 0.31 in Togo. In all countries except Togo, the cost of VAS via campaign is significantly higher than for routine distribution. When comparing age groups of 6-23 months with 24-59 months, there is little to no difference in cost per child.

Top-down costs are estimated from aggregated expenditure data and allocated across activities, such as campaign labour, programme management, training, supervision, and M&E, and some capsule procurement costs reported by implementing agencies. Bottom-up costs are estimated by identifying and costing specific inputs, such as facility-based staff time per child, vitamin A capsule unit costs multiplied by doses delivered, and other recurrent costs per visit based on standard ingredients assumptions.

**Table E-2: Baseline costs of vitamin A supplementation per child covered by service delivery modality, by country, in USD**

|  | Campaign | | | Routine | | |
| --- | --- | --- | --- | --- | --- | --- |
|  | DRC | Togo | Niger | DRC | Togo | Niger |
| Total | 2,977,944 | 126,515 | 3,837,108 | 3,131,663 | 86,603 | 169,190 |
| Target population (6-59 months) | [23,247,462](https://docs.google.com/spreadsheets/d/1xtCNCVTF6P8fDcD0J8kwfpJb8yhMyfCR/edit?gid=735017311) | [1,602,761](https://docs.google.com/spreadsheets/d/1CkuxMk72Eou2ImHvxFx3BiUjWMVDAUCuTECuAhJZd-k/edit?gid=2054611120) | [6,949,030](https://docs.google.com/spreadsheets/d/1xz8x-9LvCx5HYJZvDXfYNFRQgmkcQvD5/edit?gid=773026514) | 23,247,462 | 1,602,761 | 6,949,030 |
| Total number of children covered (6-59 months) | 5,407,469 | [1,266,723](https://docs.google.com/spreadsheets/d/1CkuxMk72Eou2ImHvxFx3BiUjWMVDAUCuTECuAhJZd-k/edit?gid=2054611120) | 6,852,876 | 13,245,179 | 283,544 | 561,564 |
| Total baseline cost per child covered (6-59 months) | 0.55 | 0.1 | 0.56 | 0.24 | 0.31 | 0.30 |
| Baseline cost per child covered (6-23 months) | 0.54 | 0.1 | 0.56 | 0.20 | 0.31 | 0.26 |
| Baseline cost per child covered (24-59 months) | 0.56 | 0.1 | 0.56 | 0.26 | 0.31 | 0.34 |

USD, United States Dollar

Source: Authors using internal documents from UNICEF DRC, UNICEF Togo, UNICEF Niger, Helen Keller International, Nutrition International, Ministry of Health, Hygiene and Prevention of Togo, Ministry of Public Health in Niger, Ministry of Health National Nutrition Program; Programme National de Nutrition (PRONANUT) DRC.

Campaign delivery is predominantly financed by donors, with government contributions accounting for no more than 0.1% of total campaign funding in all three countries (**Table D-3**). In contrast, routine distribution is primarily supported by domestic resources, with governments funding between 77.9% and 94.9% of routine delivery costs. This stark contrast underscores the reliance on external financing for campaigns and the comparatively greater domestic ownership of routine service delivery.

**Table E-3: Financing sources of vitamin A supplementation by country, by service delivery modality**

|  | Campaign | | | Routine | | |
| --- | --- | --- | --- | --- | --- | --- |
|  | DRC | Togo | Niger | DRC | Togo | Niger |
| Hellen Keller International | 82.6% | 77.71% | 90.6% |  |  |  |
| Nutritional International |  | 22.29% |  |  | 4.1% |  |
| UNICEF | 17.36% |  | 9.3% | 22.1% | 1% | 17.9% |
| Government | 0.04% | 0% | 0.1% | 77.9% | 94.9% | 82.1% |

Source: UNICEF country offices of DRC, Togo and Niger. Helen Keller International. Nutrition International. Ministry of Health, Hygiene and Prevention of Togo, Ministry of Public Health in Niger, Ministry of Health National Nutrition Program; Programme National de Nutrition (PRONANUT) DRC.

1. World Health Organization. (n.d.). Quantities and unit prices (cost inputs). *World Health Organization*. Retrieved December 17, 2024, from<https://www.who.int/teams/health-systems-governance-and-financing/economic-analysis/costing-and-technical-efficiency/quantities-and-unit-prices-(cost-inputs)> [↑](#footnote-ref-1)
2. Adjustments may be needed to reflect costs resulting from deploying CHW or other staff exclusively for VAS and/or in very remote areas where productivity of CHW may be justifiably low(er). [↑](#footnote-ref-2)
3. World Bank. (2023). *Official exchange rate (LCU per USD, period average).* Accessed 23^rd^ June 2024. Available online: https://data.worldbank.org/indicator/PA.NUS.FCRF?skipRedirection=true&view=map [↑](#footnote-ref-3)
4. World Bank. (2023). *Official exchange rate (LCU per US$, period average).* Accessed 23^rd^ June 2024. Available online: https://data.worldbank.org/indicator/PA.NUS.FCRF?skipRedirection=true&view=map [↑](#footnote-ref-4)
5. World Bank (2023). Official exchange rate (LCU per US$, period average). Accessed 23^rd^ June 2024. Available online: https://data.worldbank.org/indicator/PA.NUS.FCRF?skipRedirection=true&view=map [↑](#footnote-ref-5)
6. World Bank (2023). *Official exchange rate (LCU per US$, period average).* Accessed 23^rd^ June 2024. Available online: https://data.worldbank.org/indicator/PA.NUS.FCRF?skipRedirection=true&view=map [↑](#footnote-ref-6)
